# Role of Corticotropin Releasing Factor 1 Signaling in Cocaine Seeking during Early Extinction in Female and Male Rats

**DOI:** 10.1371/journal.pone.0158577

**Published:** 2016-06-30

**Authors:** Angie M. Cason, Amy Kohtz, Gary Aston-Jones

**Affiliations:** 1 Department of Neurosciences, Medical University of South Carolina, Charleston, South Carolina, United States of America; 2 Brain Health Institute, Rutgers University/Rutgers Biomedical and Health Sciences, Piscataway, New Jersey, United States of America; Radboud University Medical Centre, NETHERLANDS

## Abstract

Locus coeruleus norepinephrine (LC-NE) and corticotropin releasing factor (CRF) neurons are involved in stress responses, including stress’s ability to drive drug relapse. Previous animal studies indicate that female rats exhibit greater drug seeking than male rats during initial drug abstinence. Moreover, females are more sensitive to the effect of stress to drive drug seeking than males. Finally, LC-NE neurons are more sensitive to CRF in females compared to males. We hypothesized that increased drug seeking in females on extinction day one (ED1) is due to increased response to the stress of early withdrawal and is dependent upon the increased response of LC in females to CRF. We predicted that LC-NE neurons would exhibit Fos activation on ED1, and that blocking CRF1 signaling would decrease drug seeking on ED1 measured by responding on an active lever previously associated with cocaine self- administration. After chronic cocaine self-administration, female and male rats underwent a test for initial extinction responding by measuring lever pressing in the absence of cocaine. Prior to this Extinction Day 1 (ED1) session, rats were injected with vehicle or the selective CRF1 antagonist (CP) to measure effects of CRF antagonism on drug seeking during early abstinence. ED1 increased corticosterone in female rats, in proportion to lever responding in male and female, indicating that ED1 was stressful. Pretreatment with CP decreased cocaine seeking on ED1 more effectively in female compared to male rats. This increase in responding was associated with an increase in activation of LC NE neurons. Together, these findings indicate that stress, and signaling at CRF receptors in LC, may be involved in the increased drug seeking during initial abstinence.

## Introduction

Accumulating evidence indicates that there is a strong relationship between stress, substance abuse and sex [1,2]. Clinical findings indicate that there is increased neural activation in response to stressful stimuli in cocaine-dependent women compared to cocaine-dependent men, which may influence the propensity to relapse [3]. It is well known that brain norepinephrine (NE) and corticotropin releasing factor (CRF) systems play important roles in the response to stress, and recent findings indicate a link between the sex/gender differences in drug abuse and these brain systems. In particular, locus coeruleus (LC) NE neurons in females are more sensitive to stressors and CRF receptor activation than in males, and this effect is independent of estrous cycle [4].

In animal models, stress drives reinstatement of drug seeking in a CRF-dependent manner [5–8], and females show greater stress-induced reinstatement compared to males [9,10]. Notably, several studies link stress and the central NE system in relapse to drug seeking [11–15].

Although several studies have investigated the role of stress and sex in relapse to drug seeking, few studies have investigated how these factors influence drug seeking during initial abstinence or the early withdrawal period. Following a period of chronic drug intake, female rats exhibit increased drug seeking during the first day of drug abstinence compared to male rats [16–20]. We hypothesized that the first day of abstinence is stressful due to the absence of expected drug, and that the increased response of LC-NE neurons in females drives the increased stress response during initial abstinence leading to increased drug seeking in female compared to male rats. Here, we tested this hypothesis by measuring Fos activation of LC-NE neurons during the first day of extinction (ED1) after chronic cocaine self-administration, measuring corticosterone levels in males and females on ED1, and by disrupting CRF neurotransmission with a CRF antagonist on ED1. Fos activation in TH positive cells of A1 or A2/NTS, nucleus solitary tract, regions was measured for comparison.

## Methods

### Subjects

Female and male Sprague Dawley rats (Charles River, Wilmington, MA, USA) were singly housed under a reversed 12h/12h light/dark cycle (lights off 0600 h). Rats had free access to food and water and were housed in the animal facility at the Medical University of South Carolina (AAALAC-accredited). All experiments were approved by the Medical University of South Carolina’s Institutional Animal Care and Use Committee and conducted in accordance to the National Institutes of Health specifications outlined in their Guide for the Care and Use of Laboratory Animals. A mixture of ketamine and xylazine was used for anesthesia during surgery and prior to sacrificing animals via perfusion followed by decapitation and brain removal for immunochemistry.

### Jugular catheter surgeries

Animals were anesthetized with ketamine/xylazine (56.5/8.7 mg/kg) and given meloxicam (1 mg/kg) as an analgesic. Chronic in-dwelling catheters were inserted into the right jugular vein and exited the dorsal side of the body via port between the scapulae. Animals were given cefazolin (10 mg, iv) and heparin (10 U, iv) starting 3 days following surgery and daily following self-administration sessions. Rats were allowed to recover from surgery for 1 week before self-administration training.

### Fixed ratio responding for cocaine

Self-administration sessions were conducted in standard operant chambers housed in sound attenuating cubicles and controlled via MED-PC IV software (Med-Associates, St Albans, VT, USA) as described previously [21]. All rats were trained to lever press for cocaine reward (intravenous (iv) infusion of cocaine hydrochloride, 0.20mg/50 μl infusion) on a fixed ratio 1 (FR1) schedule of reinforcement during daily 2 h sessions. Presses on the inactive lever had no programmed consequences. A discrete light + tone compound cue (78 dB, 2900 Hz; white stimulus light above the active lever) accompanied the iv cocaine infusion and was followed by a 20 s timeout. The red house light (on the wall opposite the levers) was turned off during iv infusions and time-outs. Rats were trained in self-administration until they produced 10 sessions in which they earned ≥ 10 cocaine infusions.

### Extinction (cocaine-seeking) tests

24 h after the final self-administration session, rats were given interpareteonal (ip) injections of CP 154–526 (CP), CRF 1 receptor antagonist, 20 min prior to the extinction session. During the 90 min extinction session, cocaine reward and discrete cues were withheld. Presses on either lever had no consequences. 15 min following the extinction session, rats were deeply anesthetized and transcardially perfused using saline and 4% paraformaldehyde. Brains were collected, post-fixed for 24 hours in 4% paraformaldehyde, transferred to a 20% sucrose solution, and stored at 4°C. Coronal sections (40 μm thick) were cut using a cryostat and processed for single or double label immunohistochemistry (Fos and tyrosine hydroxylase, TH).

A separate group of rats were given ip injections of saline or CP 154–526 (CP) vehicle 24 h after the final self-administration session and returned immediately to their home cage. 2 h later the rats were sacrificed and brains collected for immunohistochemistry as described above.

### Fos immunohistochemistry

Sections were placed in 0.01M phosphate buffered saline (PBS) solution with 2% sodium azide added and 0.3% hydrogen peroxide for 15 min. After three 5-min washes with PBS, sections were transferred to PBS with 0.3% Triton added (PBST) containing 2% normal donkey serum (NDS) for 2 h. Sections were incubated overnight (16h) in the same solution with the addition of primary antibody (Fos antibody at 1:10,000 Calbichem, Santa Cruz, CA). Sections were rinsed three times (3x) in PBST and transferred to secondary antibody (biotinylated donkey anti-rabbit, 1:500, Jackson ImmunoResearch Laboratories, West Grove, PA) for 2 h. Next, sections were rinsed 3x in PBST and transferred to solution containing avidin-biotin complex (1:500, Vector Laboratories, Burlington, CA) for 1.5 h. Sections were rinsed twice with PBST and once with 0.05 M Tris buffer for 5 min. To visualize Fos-related antigen-positive cells, sections were placed in 3′, 3′-diaminobenzidine (DAB) with 0.0002% H_2_O_2_ and 0.6% nickel ammonium sulphate in 0.05 M Tris buffer for 4.5 min. The DAB reaction was stopped by immediately transferring sections into Tris buffer. Next, sections were rinsed 3x in PBS for 5 min and transferred to PBS-Az for 45 min.

### Tyrosine hydroxylase (TH) immunohistochemistry

Following Fos immunohistochemistry, A1/A2/NTS sections were processed for TH immunoreactivity. Sections were transferred to PBST containing 2% NDS and TH primary antibody (1:5,000, Immunostar, Hudson, WI) and incubated overnight (16 h). Sections were rinsed 3x in PBST then transferred to a secondary antibody solution (biotinylated donkey anti-goat, 1:500, Jackson ImmunoResearch Laboratories, West Grove, PA) for 2h. Sections were rinsed 3x in PBST and transferred to a solution containing avidin-biotin complex (1:500) for 1.5 h. Sections were rinsed twice with PBST and once with 0.05 M Tris buffer for 5 min. To visualize TH-positive cells, sections were placed in DAB with 0.0002% H_2_O_2_ in 0.05 M Tris buffer. The DAB reaction was stopped after 5 min by immediate transfer into Tris buffer. The sections were rinsed in PBS and mounted on gelatin-coated slides, dehydrated and coverslipped.

Fos-positive and TH-positive cells were quantified using Openlab image processing software (Improvision) on a Macintosh computer connected to a microscope and digital camera. Neurons in the LC, A1 or A2/NTS regions were photographed and images saved to the computer (Bregma: -9.84 to -10.08mm; -14.64 to -15.00mm, respectively). The number of Fos-positive neurons, TH-positive neurons, and neurons double-labeled for Fos and TH were quantified using a point-counter tool. Two or three sections per rat were counted; consecutive sections were not analyzed to prevent double counting of cells. Neurons in the left and right hemispheres were counted independently. Comparisons between groups were done by analyzing differences between the mean values for rats in each group.

### Corticosterone enzyme-linked immunosorbent assay

Whole blood samples were obtained by intravenous catheter blood-draw immediately following ED1 testing or from homecage controls. Samples were immediately placed on ice and then centrifuged at 10,000 rpm for 10 minutes at 4°C. Plasma supernatant was pipetted into clean centrifuge tubes and was stored at -80°C until ELISA processing. The ELISA kit was obtained from Enzo Life Sciences, Inc. (ADI-900-097, Farmingdale, NY), and the assay was performed per kit instructions for plasma preparations.

### Drugs

Cocaine Hydrochloride, HCl, (NIDA, Research Triangle Park, NC) was dissolved in 0.9% sterile saline. The CRF1 receptor antagonist CP 154,526 generously donated by Pfizer was suspended in 5% cremphor and sterile water; 0, 10, or 20 mg/kg was given intraperitoneally (i.p., 4 ml/kg) 20 min prior to test sessions in all experiments.

### Statistical analysis

Independent t-tests or one-way analyses of variance (ANOVAs) were used to compare differences between groups in responding or neuronal activation. Pearson product-moment correlation coefficient were used to determine linear correlations between circulating corticosterone levels and active lever pressing on ED1. Post hoc analyses were conducted using Tukey-Kramer’s multiple comparison tests. P-values less than 0.05 were considered significant.

## Results

### Effects of CP on ED1 responding

**Fig 1:**
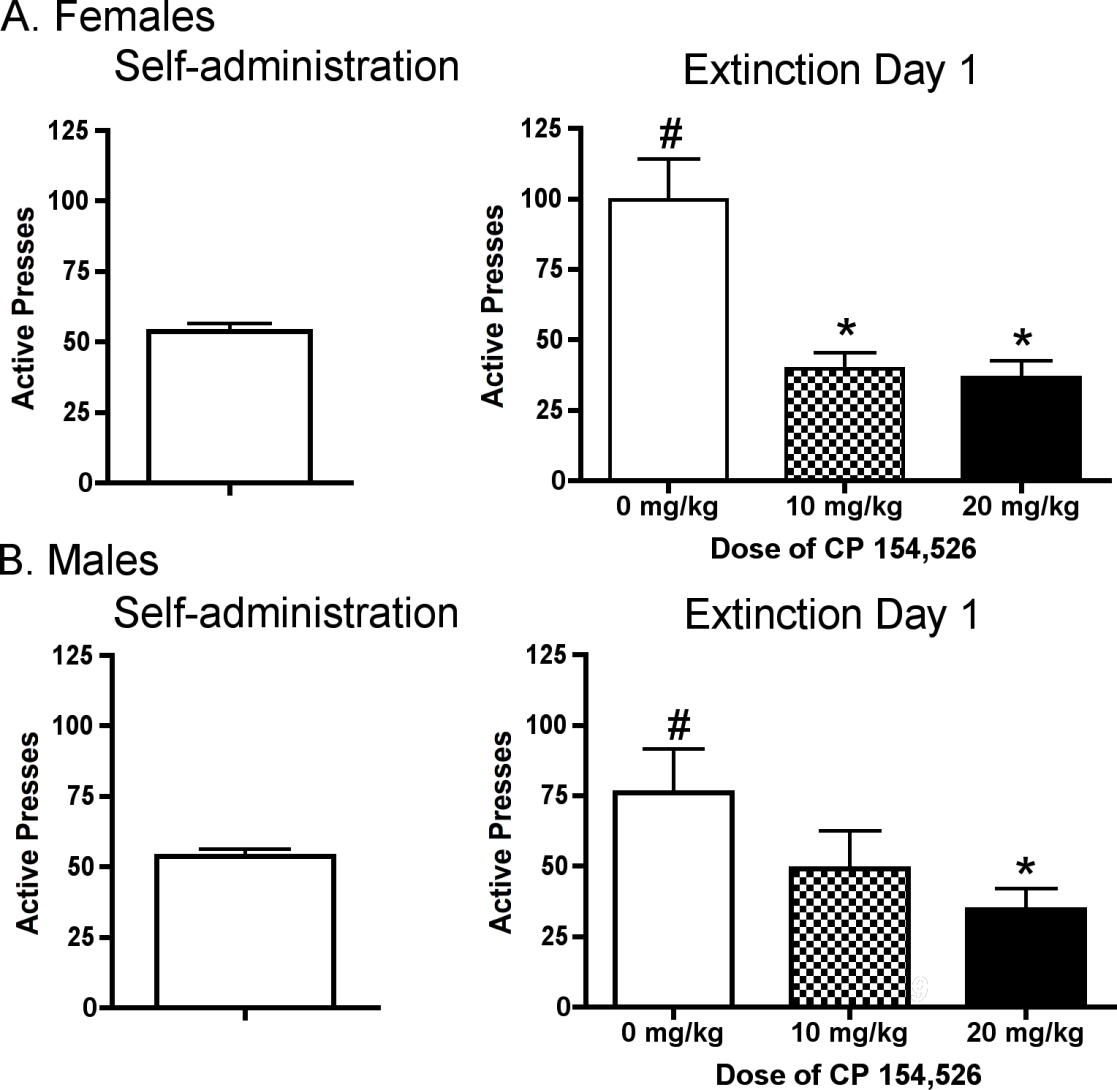
Effects of CP on ED1 Responding. Responding for cocaine was increased during initial abstinence (ED1) compared to self-administration responding in female and male rats. Pretreatment with the CRF 1 receptor antagonist (CP) attenuated ED1 responding for cocaine compared to vehicle to a greater degree in female compared to male rats. # p < 0.05 compared to self-administration; * p < 0.05 versus CP vehicle on ED1.

Female (n = 33) and male (n = 29) rats exhibited similar levels of active lever pressing during self-administration (females: 53.75 ± 3.02, males: 53.67 ± 2.72, mean ± SEM for last 3 days of self-administration; Fig 1A and 1B left panels). Active lever pressing was increased in vehicle treated controls on ED1 compared to self-administration in female rats [t(43) = 4.61, p < 0.010] and male rats [t(35) = 2.36, p < 0.05] (Fig 1). Active lever pressing appeared higher in female compared to male controls on ED1; however, independent t-test revealed that there was not a significant difference in ED1 responding in female compared to male rats. One-way ANOVAs revealed a significant effect of treatment condition (CP: 0, 10, or 20 mg/kg) in female [F(2,31) = 12.66, p < 0.001] and male rats [F(2,26) = 3.55, p < 0.05] on active pressing during ED1. Post hoc analysis revealed that both doses of CP reduced active lever pressing compared to vehicle pretreatment on the first day of extinction in females (p< 0.01 for each dose, Fig 1A, right panel), but only the highest dose of CP reduced active lever pressing compared to vehicle pretreatment on the first day of extinction in males (p < 0.05, Fig 1B, right panel).

### Fos Expression in LC: Effects of CP

**Fig 2:**
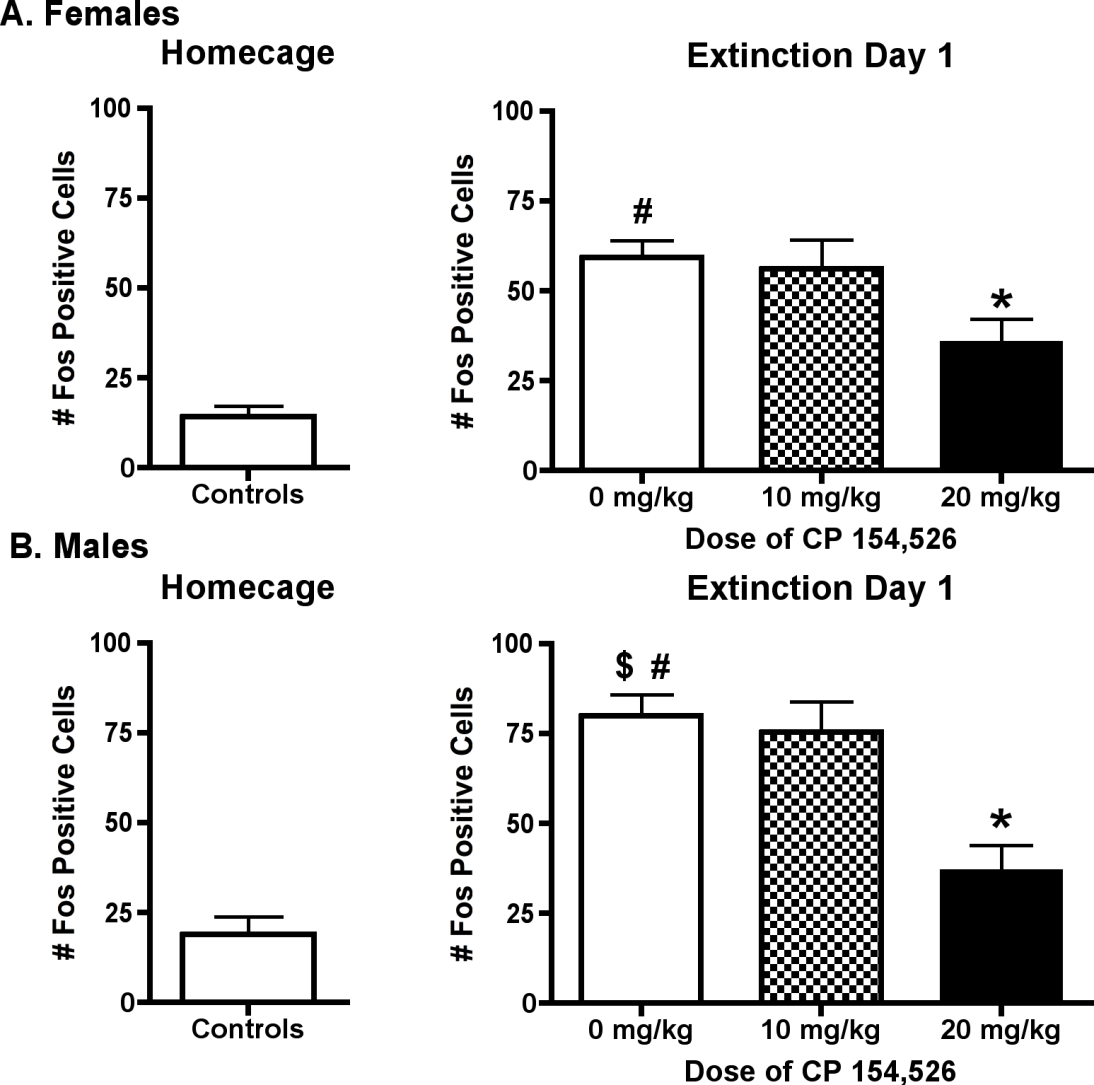
Fos Expression in Locus Coeruleus (LC) neurons. Fos expression was increased in LC neurons of vehicle treated rats during initial abstinence compared to home cage controls; CP (20 mg/kg) decreased Fos expression in LC in female and male rats. # p < 0.05 versus home cage, * p < 0.05 versus 0mg/kg on ED1, $ p <0.05 compared to females.

Given the densely packed nature of TH positive cells in LC, sections in this region were only processed for Fos immunoreactivity. The number of Fos positive nuclei in LC was increased during ED1 compared to home cage controls in female [t(30) = 8.56, p < 0.001] and male [t(26) = 7.53, p < 0.001] rats. The effects of pretreatment with CP on Fos expression in LC are illustrated in Fig 2. Separate one-way ANOVAs were used to compare the effects of CP on Fos expression in female and male rats. One-way ANOVAs revealed that pretreatment with CP decreased Fos expression in LC during ED1 in female [F(2,37) = 4.19, p < 0.05] and male rats [F(2,41) = 10.51, p < 0.001]. Post hoc analyses revealed that CP (20 mg/kg) decreased Fos expression in LC in both female and male rats (p < 0.05 for each sex).

### Fos expression in A2/NTS TH positive neurons: Effects of CP

**Fig 3:**
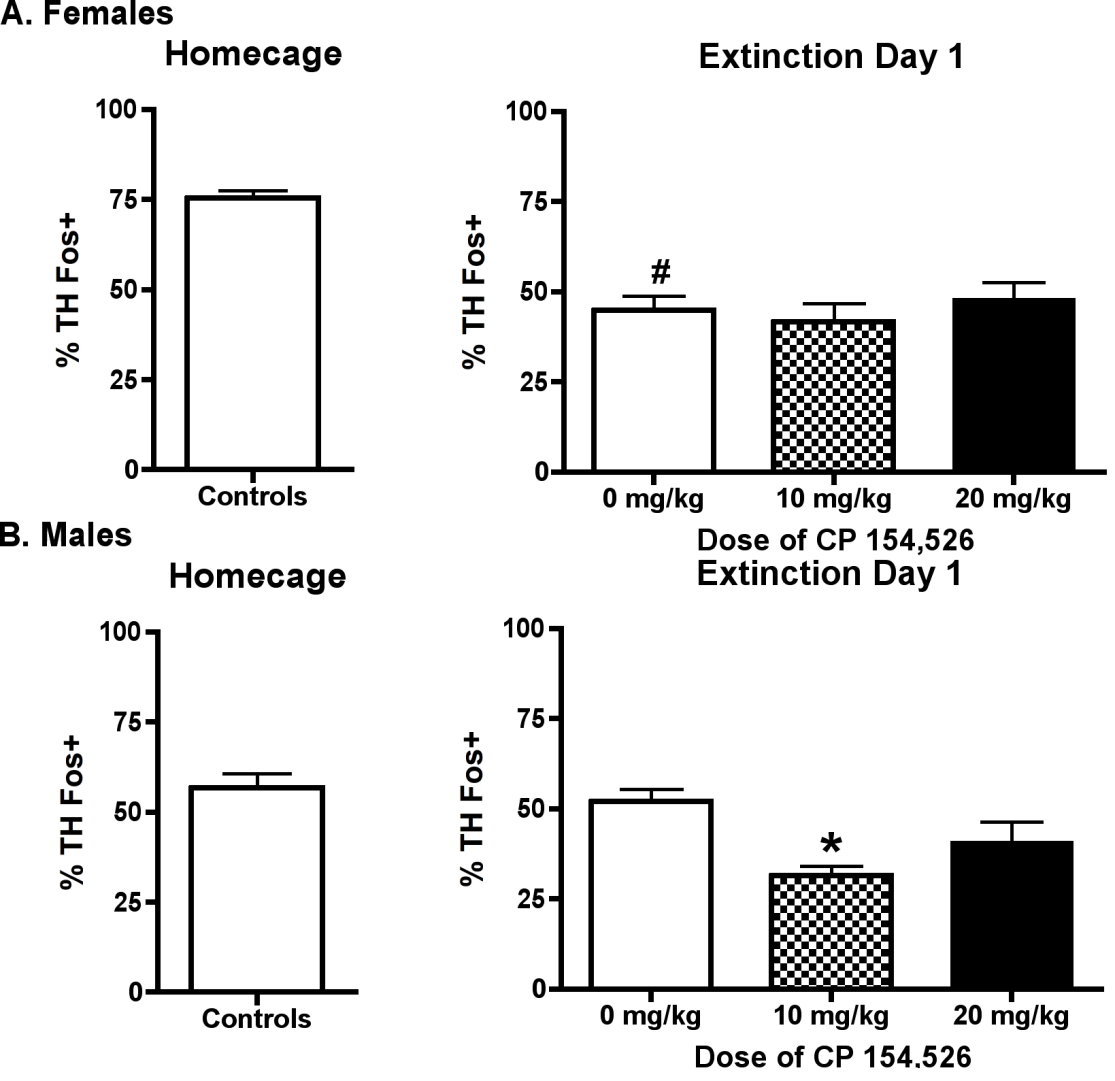
% TH neurons Fos+ in A2/NTS. The % of TH neurons Fos positive was decreased compared to home cage controls on ED1 in female, but not male, rats; CP (10 mg/kg) decreased the percentage of TH neurons Fos positive in in male, but not, female rats. # p < 0.05 versus home cage, * p < 0.05 versus 0 mg/kg on ED1.

Female and male rats had similar numbers of TH+ neurons in A2/NTS (females: 25 ± 0.8, males 27 ± 0.9, mean ± SEM. The percentage of TH neurons Fos positive in A2/NTS was decreased during ED1 compared to home cage controls in female rats [t(28) = 7.02, p <0.001); there was no difference in percentage of TH neurons Fos positive in A2/NTS between home cage controls and ED1 controls in male rats [t(24) = 0.82, p > 0.05] (Fig 3). One-way ANOVA indicated that pretreatment with CP did not significantly affect Fos expression in TH positive A2/NTS neurons on ED1 in female [F (2,39) = 0.44, p > 0.05]; however, in contrast, one-way ANOVA revealed that pretreatment with CP decreased Fos expression in TH positive neurons in male rats on ED1 [F (2,41) = 7.52, p < 0.01]. Post hoc analysis revealed that only the 10 mg/kg dose of CP reduced the percentage of TH neurons Fos positive compared to controls (p < 0.01).

### Fos expression in A1 TH positive neurons: Effects of CP

**Fig 4:**
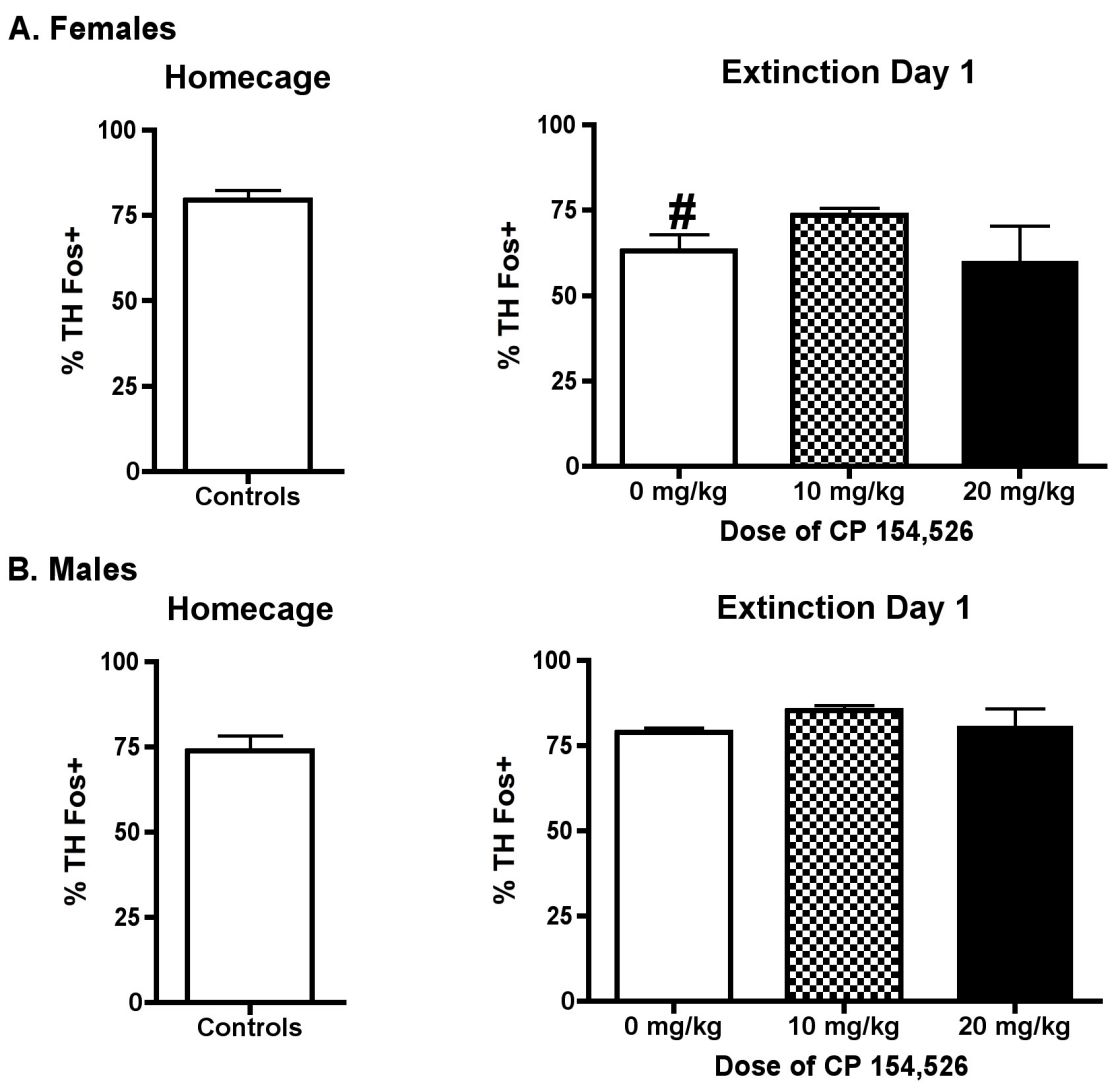
% TH neurons Fos+ in A1. The % of TH neurons Fos positive was decreased compared to home cage controls on ED1 in female rats, but not male, rats. CP had no effect on the percentage of TH neurons Fos positive on ED1 In female or male rats. # p < 0.05 versus home cage.

Female and male rats had similar numbers of TH+ neurons in A1 (females: 13 ± 0.4, males 14 ± 0.4, mean ± SEM). The percentage of TH neurons Fos positive in A1 was decreased in female controls during ED1 compared to home cage controls [t(18) = 2.56, p < 0.05); there was no difference in percentage of TH neurons Fos positive in A1 between home cage controls and ED1 controls in male rats [t(24) = 1.46, p > 0.05] (Fig 4). One-way ANOVA indicated that pretreatment with CP did not significantly affect Fos expression in TH positive A1 neurons on ED1 in female [F (2,33) = 1.46, p > 0.05] or male rats on ED1 [F (2,33) = 2.72, p > 0.05].

### Effects of ED1 exposure on plasma corticosterone levels

**Fig 5:**
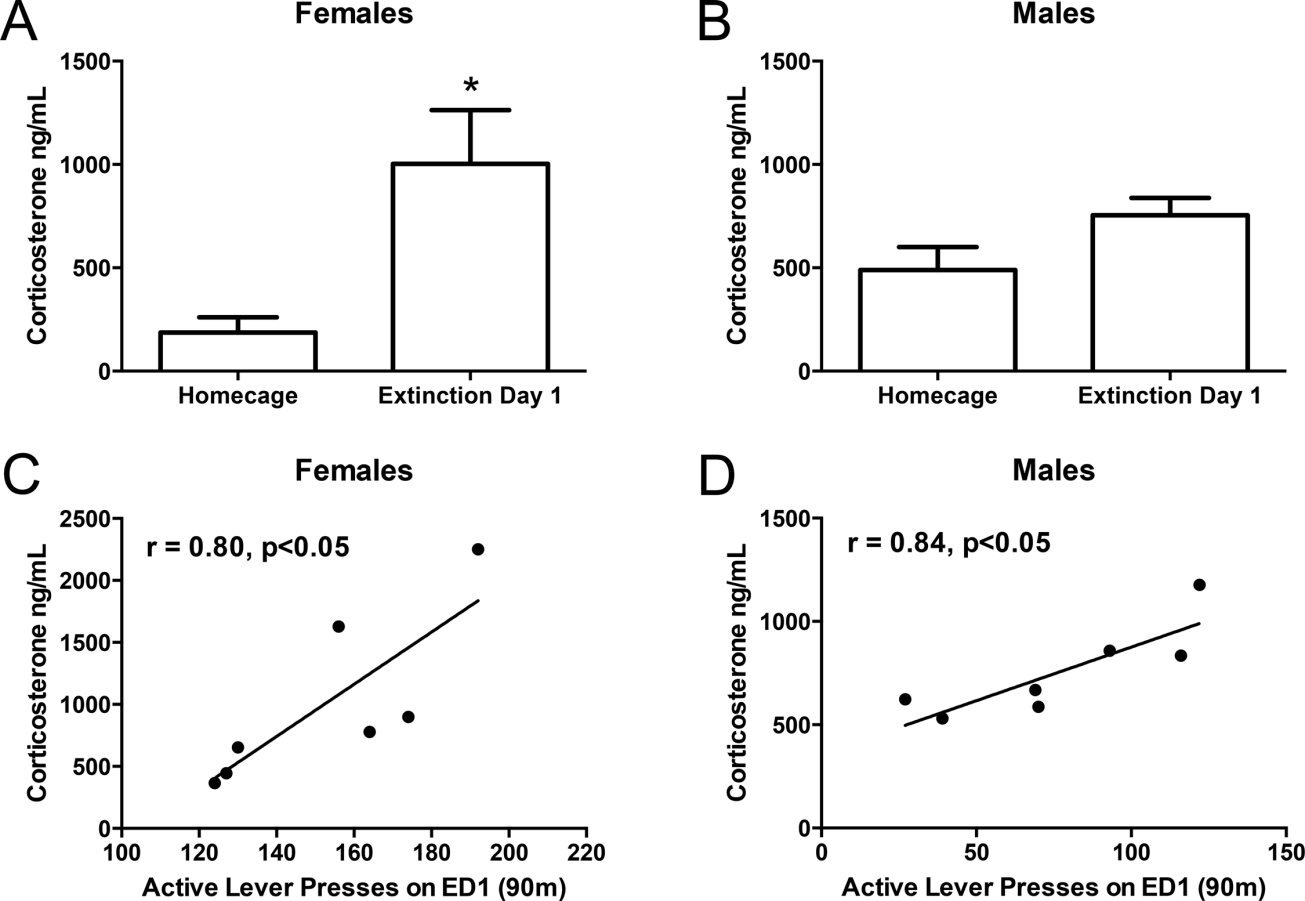
Effects of ED1 on Plasma Corticosterone Levels. Plasma corticosterone levels (ng/mL) were increased during initial abstinence (ED1) compared to homecage controls among female rats. * p < 0.05 versus homecage controls on ED1 (A). Plasma corticosterone levels significantly correlated to ED1 active lever pressing among female (C) and male (D) rats.

Female (n = 7) and male (n = 7) rats exhibited similar levels of plasma corticosterone on ED1 (females: 1003 ng/mL ± 260.6 ng/mL, males: 754.2 ng/mL ± 84.3 ng/mL, mean ± SEM). Plasma corticosterone was increased on ED1 compared to homecage in female rats [t(10) = 2.56, p < 0.05]. There was a trend for ED1 to increase plasma corticosterone compared to homecage in male rats, but the effect was not significant [t(11) = 1.93, p >0.05] (Fig 5A and 5B). A Pearson product-moment correlation coefficient was used to assess the relationship between drug-seeking behavior and plasma corticosterone levels during ED1. Drug-seeking behavior and plasma corticosterone levels were correlated on ED1 such that rats with the highest levels of ED1 responding exhibited highest corticosterone levels when subjects were collapsed across sex [r = 0.59, p < 0.05], and when analyzed separately among female [r = 0.80, p < 0.05] and male [r = 0.84, p < 0.05] rats.

## Discussion

Our results support previous findings demonstrating that rats exhibit increased cocaine seeking during initial abstinence [16–20]. Moreover, our results indicate that the increased cocaine seeking observed during initial abstinence, extinction day 1 (ED1), is accompanied by increased activation of LC-NE neurons, but not other NE neurons in A1 or A2/NTS. Pretreatment with CP decreased cocaine seeking on ED1 in female and male rats, but was more effective in females supporting the hypothesis that signaling at the CRF1 receptor is involved in drug seeking during initial abstinence. Although CP was more effective at decreasing cocaine seeking in female compared to male rats, it was equally effective at decreasing activation of LC-NE neurons in female and male rats. Some of these effects may be due to a greater stress response on ED1 observed among females, compared to males.

Together, these findings indicate that CRF signaling in LC-NE neurons may be involved in increased drug seeking during initial abstinence. As well, ED1 drug-seeking was correlated with increased circulating corticosterone levels which suggests that increases in CRF signaling in LC-NE neurons and increases plasma corticosterone levels on ED1 may be associated. It is unknown if the effects of CP on cocaine seeking and CRF signaling in LC-NE neurons on ED1 are influenced by plasma corticosterone levels. However, plasma levels of corticosterone are increased on ED1 to a greater extent among female rats compared to male rats. These data suggest that increased efficacy of CP to reduce ED1 responding among females may result from counteracting a greater stress response on ED1.

Although cocaine seeking appeared higher in females compared to males on ED1, this effect did not reach statistical significance. Our results for a sex difference in ED1 responding were not as robust as in previous reports that indicated that female rats show increased cocaine seeking during initial abstinence. This discrepancy between our findings and previous reports is most likely due to the fact that the current cohort of male rats in our study showed higher responding on the first day of extinction compared to male rats in a previous report [17]. It is unclear why males in the current study responded more than is typical on ED1.

Increased cocaine seeking on ED1 was associated with an increase in Fos expression of LC-NE neurons. We hypothesize that increased Fos expression in LC is due to increased CRF input during ED1 that in turn increases activity in LC-NE neurons. In support of this hypothesis, CRF strongly activates NE neurons in LC [22–24]. Moreover, previous findings from our laboratory indicate that systemic clonidine, an α2 autoreceptor agonist decreases NE release, decreases responding when given on the first day of extinction, and systemic α1 and β-adrenoceptor antagonists block stress-induced reinstatement of cocaine seeking [21]. We do not believe that the increase in Fos expression during ED1 is due to increased locomotor activity or that CP effects on Fos expression are due to general suppression of locomotor activity. Findings from Hearing et al. 2008 indicate that increased immediate early gene expression during relapse to cocaine seeking is independent of lever pressing, and CP does not affect basal locomotor activity levels or alter the rewarding effects of cocaine during self-administration [25,26]. Although we speculate that the effects in observed in LC-NE neurons is due to CRF in LC, CRF in other brain regions that innervate LC may be involved in these effects.

The finding that CP decreased cocaine seeking during initial abstinence more effectively in female than male rats despite having similar effects on LC Fos expression suggests that CRF signaling outside LC may also play a role in the observed sex difference. The medial prefrontal cortex is a likely candidate given that it receives a strong NE input from LC and has been previously implicated in reinstatement models of drug seeking [27–32]. Inhibition of PFC attenuates cue- and cocaine-induced reinstatement [32,33]. In addition, BNST is a region rich in CRF receptors that has been previously implicated in stress-induced reinstatement of drug seeking, and reported to play a role in increased drug seeking during abstinence [34,35]. Our findings that Fos expression was not increased in NTS NE neurons on the first day of extinction appears to rule out that as a NE pathway involved in ED1 responding. Lastly, we cannot rule out effects via interactions with the oxytocin (OT) system. OT neurons are involved in reward and anti-stress processes, perhaps via interactions with NE and CRF, and play a role in driving drug craving [36–38]. Indeed, findings from several recent studies indicate that oxytocin decreases cocaine seeking and economic demand for cocaine in rats [39–41].

In conclusion, our findings demonstrate that signaling at the CRF1 receptor is involved in cocaine seeking during initial abstinence in female and male rats, and that CP, the CRF1 receptor antagonist, is more effective at attenuating cocaine seeking in female compared to male rats. Furthermore, increased cocaine seeking during initial abstinence is associated with increased activation of LC-NE neurons and release of corticosterone, indicating that an interaction between the CRF and NE systems regulate cocaine seeking. Together, our findings suggest that initial abstinence may represent an important time point for attenuating drug seeking using pharmacological interventions. A clearer understanding of how CRF, NE and OT are involved in sex/gender-specific drug seeking may lead to more effective treatments for addiction in both males and females.
